# Associations Between Online Pornography Consumption and Sexual Dysfunction in Young Men: Multivariate Analysis Based on an International Web-Based Survey

**DOI:** 10.2196/32542

**Published:** 2021-10-21

**Authors:** Tim Jacobs, Björn Geysemans, Guido Van Hal, Inge Glazemakers, Kristian Fog-Poulsen, Alexandra Vermandel, Stefan De Wachter, Gunter De Win

**Affiliations:** 1 Faculty of Medicine and Health Sciences University of Antwerp Antwerp Belgium; 2 Department of Social Epidemiology and Health Policy University of Antwerp Antwerp Belgium; 3 Department of Urology Hospital of Holstebro Holstebro Denmark; 4 Department of Urology University Hospital Antwerp Edegem Belgium

**Keywords:** erectile dysfunction, ED, pornography, pornography-induced erectile dysfunction, PIED, sexual health, young adults

## Abstract

**Background:**

Expanding access to the internet has resulted in more and earlier consumption of online pornography. At the same time, a higher prevalence of erectile dysfunction (ED) among young men is seen. Increased pornography consumption has been suggested as a possible explanation for this rise.

**Objective:**

The aim of this study was to better understand associations between problematic pornography consumption (PPC) and ED.

**Methods:**

A 118-item survey was published online, and data collection took place between April 2019 and May 2020. Of the 5770 men who responded, the responses from 3419 men between 18 years old and 35 years old were analyzed. The survey used validated questionnaires such as the Cyber Pornography Addiction Test (CYPAT), International Index of Erectile Function (IIEF-5), and Alcohol Use Disorders Identification Test-Concise (AUDIT-C). The estimated amount of porn watching was calculated. Univariable and multivariable analyses were performed. For the multivariable analysis, a logistic regression model using a directed acyclic graph was used.

**Results:**

According to their IIEF-5 scores, 21.48% (444/2067) of our sexually active participants (ie, those who attempted penetrative sex in the previous 4 weeks) had some degree of ED. Higher CYPAT scores indicating problematic online pornography consumption resulted in a higher probability of ED, while controlling for covariates. Masturbation frequency seemed not to be a significant factor when assessing ED.

**Conclusions:**

This prevalence of ED in young men is alarmingly high, and the results of this study suggest a significant association with PPC.

**Trial Registration:**

Research Registry researchregistry5111; https://tinyurl.com/m45mcaa2

## Introduction

As we cannot ignore the presence of the internet (and therefore also the presence of explicit sexual materials) in the lives of young people, the effects of pornography consumption on mental and sexual well**-**being are often questioned.

Several reports have underlined positive effects of pornography consumption (more sexual comfort and self-acceptance, lower levels of shame and anxiety towards personal sexual orientation, increased interest in sex and sexual experimentation, relational happiness, more acceptance towards different sexual activities) [1-4]. Others express concern about the negative effects of pornography on sexual desire and sexual functions. However, studies looking into the impact of the frequency of pornography use on sexual functioning have not come up with consistent associations [4,5]. Still, personal and media reports (eg, [6,7]) and even TED talks (eg, [8]) claim that pornography consumption has an important effect on erectile dysfunction (ED). Even a specific term, porn-induced erectile dysfunction (PIED), was introduced [1,9,10]. Given that pornography is often accompanied by masturbation, PIED is questioned and criticized by some sexologists stating that the frequency and duration of masturbation are the key factors contributing to these negative effects and not the pornography consumption itself [11,12].

What cannot be denied is that expanding access to the internet has resulted in more and earlier consumption of online pornography. Between 50% and 70% of adult men use pornography on a regular basis, and for adolescent lifetime use, the numbers are even higher than 80% [13,14]. Simultaneously, the reported prevalence of ED in young men has increased enormously over the last decades, from 2%-5% in 1999 and 2002 to 20%-30% in more recent reports [15-18].

Getting an erection is, of course, a process that requires the neurological, hormonal, and circulatory systems to work together. Inability to get or maintain an erection can be due to penile (vascular or neurological) problems or to more centralized issues (eg, depression, lack of desire, anxiety), but these conventional components cannot entirely explain the higher prevalence of ED in young men [1]. While men aged over 50 years are more prone to have a physical cause for their ED, younger men are more likely to have a psychological cause such as performance anxiety, depression, anxiety, or relational problems [19]. There is no obvious explanation for the high prevalence of ED in younger men these days, and additionally, some patients are resistant to traditional therapy [9]. Some patients actively consult health care professionals convinced they are experiencing sexual difficulties due to their pornography consumption [20].

Certain scholars attribute these self-perceived difficulties to moral incongruence towards pornography consumption, and some link it to addiction, while others are more critical and question whether addiction models are applicable based on findings from neuroscientific studies [21-23]. Furthermore, sex addiction, porn addiction, and porn-induced sexual dysfunctions are not recognized as diagnosable entities in the 5th edition of the Diagnostic and Statistical Manual of Mental disorders (DSM-5). Patients with self-perceived porn-induced sexual problems often find themselves in a vicious circle and may struggle to find appropriate help. Individuals with problematic pornography consumption (PPC) may use pornography frequently but frequency of pornography consumption may not always be problematic [4]. As long as a theoretical framework is missing, diagnosing this entity and developing a treatment algorithm are difficult. The new International Classification of Diseases, 11th Revision (ICD-11) may bring some change for these patients as it has added compulsive sexual behavior disorder (CSBD) as a diagnosable entity. However, including CSBD in the ICD-11 did not happen without controversy [24,25]. There are many potentially dysregulated sexual behaviors that may lead to this diagnosis, but one may argue that online pornography use will be one of the most encountered in clinical settings [26]. Rather than approaching it via an addiction framework, PPC is seen as an impulse control disorder and as such might have an impact on a person’s sexual pleasure [4]. Although not completely fitting with the CSBD criteria, several screening instruments for problematic pornography use were developed and validated, making it possible to assess PPC in a more structured way [27].

A better understanding of the associations between PPC and ED might add new insights in the prevention and treatment of ED, especially in young men. These associations are complex and can only be fully understood in a multidisciplinary setting, as they require knowledge from different research fields (eg, psychology, sexology, sociology, urology) [28,29]. The aim of this study was to evaluate the frequency of pornography consumption and PPC in young men and to better understand their connection with ED.

## Methods

We organized 4 brainstorming meetings between a urologist and 2 medical practitioners with an interest in sexual health. First, a framework was designed identifying variables (including pornography consumption) that could possibly contribute to ED in young men. After a literature review by the team members, several indicators linked to pornography consumption (eg, frequency of use, age at start of use, problematic use) were listed in another brainstorming meeting. A literature review was performed identifying validated scales to measure our outcome and exposure variables, and based on scientific evidence available at that time, certain questionnaires were chosen. Then, a 118-item questionnaire was developed including questions on demography, medical history, alcohol and drug usage, sexual preferences, ED, masturbation, pornography consumption, and partner satisfaction using validated scales such as the Cyber Pornography Addiction Test (CYPAT), International Index of Erectile Function (IIEF-5), and Alcohol Use Disorders Identification Test-Concise (AUDIT-C) [30-35].

We used the definition of pornography as defined by Kraus et al [30], as “any material designed to cause or enhance sexual arousal or sexual excitement in the viewer. Such materials show clear and explicit sexual acts such as vaginal intercourse, anal intercourse, oral sex, group sex etc. Pornography does not include materials such as underwear catalogs or materials containing men and women posing naked unless these images portray clear and explicit sexual acts.”

CYPAT was included to measure the exposure, PPC. It is composed of 11 items scored on a 5-point Likert scale. Higher sum scores (minimum=11, maximum=55) indicate more problematic behavior [33]. While initially developed to screen for pornography “addiction,” it is conceived as a reliable instrument with robust psychometric properties to screen for PPC. Strong correlations with other instruments were documented, providing evidence for criterion-related validity and convergent validity [36]. CYPAT deeply measures conflict parameters and includes a use despite harm component, which is a CBSD criterion.

Outcome (ED) was measured using the IIEF-5 questionnaire, composed of 5 questions scored on a Likert scale focusing on erectile function and intercourse satisfaction. The possible sum scores for the IIEF-5 range from 5 to 25, and ED was classified into 2 categories based on the sum scores: ED (5-21) versus no ED (22-25) [31,37,38]. The IIEF-5 meets psychometric criteria for structural validity, test-retest reliability, construct validity, and criterion validity [39]. It can also be assessed online [40]. Scores on the IIEF-5 in this study were only reported for those who were sexually active in the last 4 weeks.

After a thorough review by the authors, an online, English, web-based survey was created using the Qualtrics platform and tested several times by the team members. One of the drawbacks of a web-based survey is that due to the possible nonrepresentative nature of the internet-based study sample, it can be difficult to draw population-based conclusions [41]. However, our aim was not to study the incidence of (problematic) pornography consumption or erectile dysfunction. We wanted to pilot test if and how pornography consumption correlates with sexual functioning in young men. Due to the personal and sensitive questions, the huge variation in pornography consumption and masturbation frequency in young men, and the possible small percentage of young men experiencing problematic consumption, a large sample of participants was necessary. This motivated our choice for a web-based survey as those surveys are more inclusive than postal or phone surveys allowing us to find a reasonable number of respondents with different pornography consumption habits and willing to answer these sensitive personal questions. Furthermore, once set up, a web-based survey is easy to carry out, is cheap, and creates an easy way to reach many respondents. The data are captured directly in electronic format making analysis faster and easier [42]. In the 18-35-year-old age group, it can be expected that almost everyone has access to the internet, so that the risk of selection bias by having no internet access is insignificant. We further tried to reduce selection bias by announcing the questionnaire as a survey to study current sexual health in young men without specifically mentioning this questionnaire was surveying pornography consumption.

The link to the questionnaire [43] was spread mainly in Belgium and Denmark through (social) media, posters, and flyers. Data were collected between May 2019 and March 2020. No specific sample size was calculated as we tried to reach as many participants as possible. All participants signed informed consent before participating. The Qualtrics settings allowed us to avoid duplicate entries. This research adhered to the latest version of the Declaration of Helsinki and was approved by the ethics committee for the Social Sciences and Humanities of the University of Antwerp. All data were anonymized and nonidentifiable.

To evaluate the association between PPC and ED in those who had penetrative sex in the previous 4 weeks, the initial research group teamed up with a psychologist, a sociologist, and an epidemiologist to participate in multidisciplinary brainstorming sessions to conceptualize the exposure-outcome relationship between PPC and ED. A directed acyclic graph (DAG) was used to visualize the associations between the covariates and CYPAT and ED and to classify the covariates as confounders (common causes of CYPAT and ED), mediators (covariates on the causal pathway from CYPAT to ED), and colliders (CYPAT and ED independently cause a third variable). DAGs have become an established framework for the analysis of causal inference in epidemiology and are used to show how associations translate into causal relations [44,45]. For this study, a DAG was used a posteriori to examine potential causality of PPC on ED and to guide the multivariable data analysis.

Descriptive statistics summarizing and describing the characteristics of the data (eg, demographics, sexual interests, masturbation frequency, PPC) were performed both for those men who had penetrative sex during the preceding 4 weeks and those who had not. The pornography consumption time (PCT) in minutes per week was calculated post hoc based on frequency of masturbation, the number of times pornography was used for masturbation, and the average length of 1 pornography session.

Nonparametric tests (chi-square, Kruskal Wallis, and Mann-Whitney-U) were used for the univariate analyses. A significance level of .05 was used to determine statistical significance.

The minimal sufficient adjustment sets for estimating the direct effect of PPC on ED were identified using the DAGitty v3.0 web application and included in a multivariable regression analysis [45]. The following 12 covariates were identified as potential confounders, mediators, or colliders: masturbation frequency, relationship status, partner satisfaction, substance abuse, somatic causes, libido, depression, use of antidepressants, exercise, sexual identity, real sex vs pornography preference, and performance pressure. Statistical analyses must account for confounding factors, without introducing bias when controlling for variables that are not on the causal path between the exposure and the outcome. DAGgity software was used to identify the minimal sufficient adjustment set. Assuming this DAG is plausible, a minimal sufficient adjustment set for estimating the direct effect of PPC on ED consisted of 8 covariates: identity, libido, masturbation frequency, relationship status, use of antidepressants, arousal (real sex vs pornography), partner satisfaction, and performance pressure. These covariates were included in a logistic regression model.

This multivariable logistic regression model was constructed to estimate the effect of the selected covariates. Odds ratios (OR) and corresponding 95% CIs were calculated to evaluate the strength of the associations. All statistical analyses were performed using R software v4.02, RStudio v 1.3.959, and Jamovi v1.8.

## Results

### Sample Characteristics

**Figure 1:**
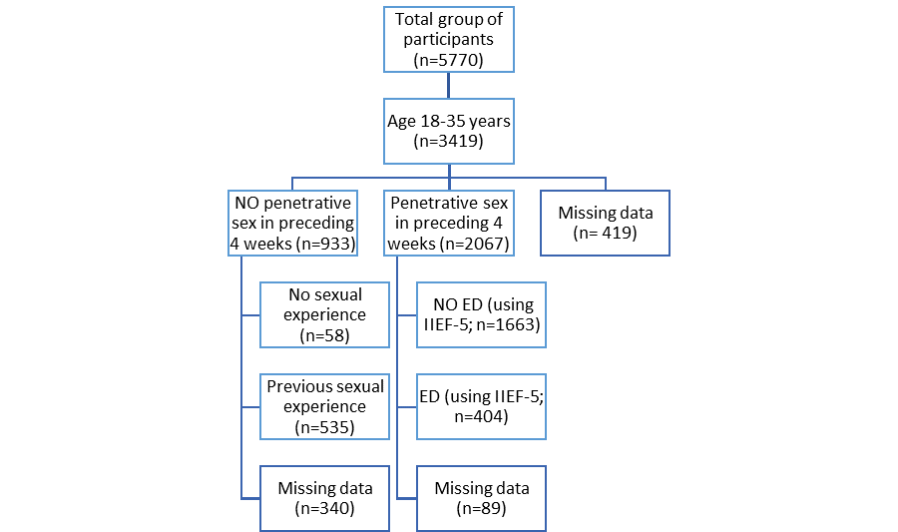
Flowchart for participant selection. ED: erectile dysfunction; IIEF-5: International Index of Erectile Function, Short version.

A total of 5770 men responded to our questionnaire spread mainly but not exclusively via newspaper or radio (2423/5770, 41.99%), social media (1789/5770, 31.00%), and student mailing (577/5770, 10.00%); 2351 participants (2351/5770, 40.75%) were excluded because they were over 35 years of age. Median time to complete the questionnaire was 20 minutes. Eventually, the results of 3419 participants were analyzed. As IIEF-5 asks questions specifically about problems during penetrative sexual intercourse, the participants were divided in 2 categories: those who had penetrative sex in the previous 4 weeks and those who did not. See Figure 1 for a flowchart of our participant selection. Demographics based on these 2 categories can be found in Table 1.

**Table 1:** Demographics.

Characteristics		Penetration attempt in the past 4 weeks?			Overall (n=3419)
		No (n=933)	Yes (n=2067)		
Age (years), median (minimum-maximum)		22.0 (2.00-35.0)	25.0 (0.340-35.0)	24.0 (0.340-35.0)	
**Educational level, n (%)**					
	Less than high school diploma	34.0 (3.6)	57.0 (2.8)	112 (3.3)	
	High school diploma or equivalent degree	387 (41.5)	545 (26.4)	1093 (32.0)	
	Bachelor’s degree	320 (34.3)	808 (39.1)	1278 (37.4)	
	Master’s degree	183 (19.6)	606 (29.3)	865 (25.3)	
	Doctorate	9 (1.0)	51 (2.5)	71 (2.1)	
**Relationship status, n (%)**					
	Single	814 (87.2)	368 (17.8)	1340 (39.2)	
	In a “new” relationship (<6 months)	28 (3.0)	201 (9.7)	277 (8.1)	
	In a longstanding relationship (>6 months)	74 (7.9)	1107 (53.6)	1327 (38.8)	
	Engaged or married	15 (1.6)	387 (18.7)	435 (12.7)	
	Divorced or widowed	2 (0.2)	4 (0.2)	9 (0.3)	
	Missing	0 (0)	0 (0)	31 (0.9)	
“**Do you smoke?”, n (%)**					
	No, I never smoked	644 (69.0)	1162 (56.2)	1981 (57.9)	
	No, but I did smoke in the past	94 (10.1)	356 (17.2)	502 (14.7)	
	Yes, but only occasionally	108 (11.6)	321 (15.5)	477 (14.0)	
	Yes	87 (9.3)	228 (11.0)	349 (10.2)	
	Missing	0 (0)	0 (0)	110 (3.2)	
AUDIT-C^a^ score, median (minimum-maximum)		5.00 (1.00-12.0)	5.00 (1.00-12.0)	5.00 (1.00-12.0)	
Missing AUDIT-C values, n (%)		133 (14.3)	117 (5.7)	442 (12.9)	
**“In the past 2 weeks, have you frequently been hindered by depressive feelings or feelings of helplessness?”, n (%)**					
	No	573 (61.4)	1661 (80.4)	2393 (70.0)	
	Yes	360 (38.6)	406 (19.6)	822 (24.0)	
	Missing	0 (0)	0 (0)	204 (6.0)	
**“I identify myself as”, n (%)**					
	Heterosexual only	631 (67.6)	1477 (71.5)	2235 (65.4)	
	Heterosexual mostly	122 (13.1)	302 (14.6)	446 (13.0)	
	Heterosexual, somewhat more bisexual	36 (3.9)	83 (4.0)	126 (3.7)	
	As equally heterosexual as homosexual	16 (1.7)	25 (1.2)	46 (1.3)	
	Homosexual, somewhat more bisexual	18 (1.9)	14 (0.7)	32 (0.9)	
	Homosexual mostly	33 (3.5)	47 (2.3)	80 (2.3)	
	Homosexual only	64 (6.9)	112 (5.4)	184 (5.4)	
	Asexual	8 (0.9)	2 (0.1)	10 (0.3)	
	Missing	0 (0)	0 (0)	247 (7.2)	
**“I am attracted to”, n (%)**					
	Women only	653 (70.0)	1536 (74.3)	2322 (67.9)	
	Women mostly	112 (12.0)	266 (12.9)	396 (11.6)	
	Women somewhat more than men	25 (2.7)	51 (2.5)	80 (2.3)	
	Men and women equally	12 (1.3)	20 (1.0)	37 (1.1)	
	Men somewhat more than women	21 (2.3)	18 (0.9)	40 (1.2)	
	Men mostly	38 (4.1)	46 (2.2)	85 (2.5)	
	Men only	70 (7.5)	123 (6.0)	202 (5.9)	
	Missing	0 (0)	0 (0)	247 (7.2)	
“**My sexual fantasies are about”, n (%)**					
	Women only	614 (65.8)	1439 (69.6)	2182 (63.8)	
	Women mostly	118 (12.6)	303 (14.7)	437 (12.8)	
	Women somewhat more than men	33 (3.5)	67 (3.2)	107 (3.1)	
	Men and women equally	24 (2.6)	45 (2.2)	70 (2.0)	
	Men somewhat more than women	23 (2.5)	26 (1.3)	54 (1.6)	
	Men mostly	40 (4.3)	56 (2.7)	101 (3.0)	
	Men only	74 (7.9)	119 (5.8)	200 (5.8)	
	Missing	0 (0)	0 (0)	247 (7.2)	
CYPAT^b^ score, median (mininum-maximum)		18.0 (11.0-52.0)	16.0 (11.0-55.0)	17.0 (11.0-55.0)	
**CYPAT categories**					
	11-13	151 (16.2)	464 (22.4)	617 (18.0)	
	13-16	134 (14.4)	360 (17.4)	494 (14.4)	
	16-21	175 (18.8)	390 (18.9)	565 (16.5)	
	21-55	208 (22.3)	381 (18.4)	589 (17.2)	
	Missing	265 (28.4)	472 (22.8)	1154 (33.8)	
IIEF^c^ score, mean (SD)		N/A^d^	22.8 (2.78)	22.7 (2.97)	
IIEF score, median (minimum-maximum)		N/A	24.0 (6.00-25.0)	24.0 (5.00-25.0)	
**IIEF categories**					
	No ED^e^	N/A	1474 (71.3)	1523 (44.5)	
	Mild ED	N/A	327 (15.8)	349 (10.2)	
	Mild-moderate ED	N/A	63 (3.0)	74 (2.2)	
	Moderate ED	N/A	11 (0.5)	14 (0.4)	
	Severe ED	N/A	3 (0.1)	6 (0.2)	
	Missing	N/A	189 (9.1)	1453 (42.5)	
“How nervous are you to have any kind of sexual contact?”, median (minimum-maximum)		6.20 (0-10.0)	1.10 (0-10.0)	2.00 (0-10.0)	
Missing values for “How nervous are you to have any kind of sexual contact?”, n (%)		330 (35.4)	295 (14.3)	1043 (30.5)	
“Do you sometimes feel enormous pressure to perform in bed or to keep an erection while having sex?”, median (minimum-maximum)		6.00 (0-10.0)	2.00 (0-10.0)	3.00 (0-10.0)	
Missing values for “Do you sometimes feel enormous pressure to perform in bed or to keep an erection while having sex?”, n (%)		443 (47.5)	285 (13.8)	1146 (33.5)	
**“At which age did you first start masturbating?” (years), n (%)**					
	<10	38.0 (4.1)	131 (6.3)	169 (4.9)	
	10-12	323 (34.6)	773 (37.4)	1098 (32.1)	
	13-14	314 (33.7)	748 (36.2)	1062 (31.1)	
	15-17	57 (6.1)	137 (6.6)	194 (5.7)	
	≥18	12 (1.3)	9 (0.4)	21 (0.6)	
	Missing	189 (20.3)	269 (13.0)	875 (25.6)	
**“At which age did you start masturbating to porn?” (years), n (%)**					
	<10	7 (0.8)	20 (1.0)	27 (0.8)	
	10-12	126 (13.5)	303 (14.7)	429 (12.5)	
	13-14	350 (37.5)	851 (41.2)	1202 (35.2)	
	15-17	218 (23.4)	536 (25.9)	755 (22.1)	
	≥18	32 (3.4)	74 (3.6)	106 (3.1)	
	Missing	200 (21.4)	283 (13.7)	900 (26.3)	
**“How often do you normally masturbate?”, n (%)**					
	Regularly more than once a day	104 (11.1)	138 (6.7)	242 (7.1)	
	(Almost) every day	282 (30.2)	516 (25.0)	798 (23.3)	
	A few times a week, not every day	280 (30.0)	756 (36.6)	1038 (30.4)	
	A few times a month	26 (2.8)	143 (6.9)	169 (4.9)	
	Once a week	43 (4.6)	173 (8.4)	216 (6.3)	
	Once a month	2 (0.2)	32 (1.5)	34 (1.0)	
	Less than once a month	4 (0.4)	27 (1.3)	31 (0.9)	
	Never	3 (0.3)	13 (0.6)	16 (0.5)	
	Missing	189 (20.3)	269 (13.0)	875 (25.6)	
PCT^f^, median (minimum-maximum)		47.3 (0-1580)	35.0 (0-1560)	39.4 (0-1580)	
Missing PCT values, n (%)		220 (23.6)	361 (17.5)	998 (29.2)	
**PCT categories (minutes), n (%)**					
	0-12	106 (11.4)	438 (21.2)	545 (15.9)	
	12-35	148 (15.9)	433 (20.9)	582 (17.0)	
	35-73	196 (21.0)	408 (19.7)	604 (17.7)	
	73-1575	263 (28.2)	427 (20.7)	690 (20.2)	
	Missing	220 (23.6)	361 (17.5)	998 (29.2)	

^a^AUDIT-C: Alcohol Use Disorders Identification Test-Concise.

^b^CYPAT: Cyber Pornography Addiction Test.

^c^IIEF: International Index of Erectile Function.

^d^N/A: not applicable.

^e^ED: erectile dysfunction.

^f^PCT: pornography consumption time.

### Descriptive and Univariate Analyses

#### Masturbation

With 84.91% (2160/2544) of the participants starting masturbating between the ages of 10 years and 14 years, masturbation was a common practice in our study population. Most of our participants masturbated multiple times per week, with more than 70% (1836/2544, 72.17%) masturbating between a few times a week to daily and 9.51% (242/2544) masturbating even regularly, at more than once a day. Those who were not sexually active in the past 4 weeks seemed to masturbate more often (*P*<.001). While 89.5% (666/744) of single men masturbated multiple times a week or more, 78.42% (1410/1798) of single men in the sexually active group masturbated at the same level (*P*<.001). People in new relationships (<6 months of duration) seemed to masturbate the least (*P*<.001).

#### Pornography Consumption

Of our study participants, 98.98% (2518/2544) had consumed pornography during masturbation. Pornography was consumed during a median 8.4 of 10 masturbation sessions. In fact, 17.70% (441/2492) of our study population never masturbated without pornography consumption, and 91.40% (2222/2431) of viewers skipped to the best parts of the videos they watched. Table 2 shows the reasons for watching, where they watched, and with whom they watched.

**Figure 2:**
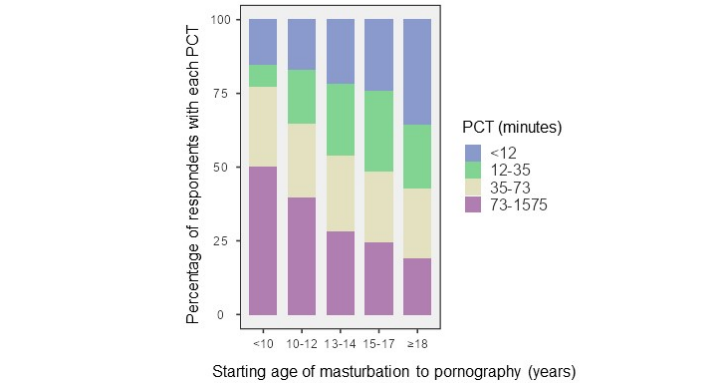
Correlations between pornography consumption time (PCT; minutes/week) and age at which masturbation to pornography started.

The median age at which the participants started masturbating to porn was 13-14 years in our population. The average porn session lasted between 5 minutes and 30 minutes in 81.50% (1973/2421) of our population. For 11.03% (267/2421), when they masturbated to pornography, it lasted, on average, more than 30 minutes. The median calculated PCT was 39.4 minutes per week. There was a statistically significant difference (*P*<.001) between those that had penetrative sex in the past 4 weeks (median 35 minutes per week) and those who had not (median 47.3 minutes per week). The starting age correlated with PCT (*P*<.001; Figure 2).

**Table 2:** Reasons for watching sexually explicit material, where they watched, and with whom they watched.

Responses		n (%)
**Reasons for watching pornography (n=3729)**		
	Because I’m horny	3145 (84.33)
	Stress relieve	1994 (53.47)
	To produce arousal for masturbating	1932 (51.81)
	Lack of real sexual contact	1597 (42.82)
	Out of boredom	1548 (41.51)
	Out of habit	1253 (33.60)
	To be able to experience fantasies or things not done/forbidden in real life	1229 (32.96)
	Sexual development/learning how to...	819 (21.96)
	Because masturbating without porn isn’t arousing enough	757 (20.30)
	To spice things up with sexual partner(s) (watching together)	740 (19.84)
	Other	108 (2.90)
**With whom they watched pornography (n=3739)**		
	Alone	3662 (97.94)
	With sexual patner(s)	1601 (42.81)
	With friend(s)	1022 (27.33)
	With stranger, online date, webcam	358 (9.57)
**Where they watched pornography (n=3741)**		
	At home/apartment/bedroom	3726 (99.60)
	Public places/toilets	1137 (30.39)
	Work	1034 (27.64)
	Friend's house/apartment	859 (22.96)
	Romantic partner’s home	857 (22.91)
	Others (eg, stranger’s house)	591 (15.80)

#### Problematic Pornography Consumption (PPC)

**Figure 3:**
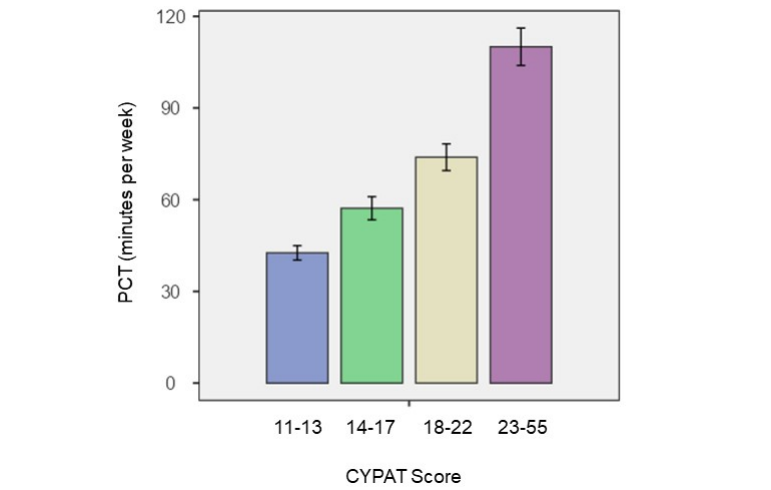
Correlation between Cyber Pornography Addiction Test (CYPAT) scores and pornography consumption time (PCT; minutes).

As there is no defined cutoff score for CYPAT, we divided the scores of our participants into quartiles, according to the distribution of the CYPAT scores [46]: 27.18% (615/2263) had a CYPAT score in Q1 (11-13 points), 27.31% (618/2263) in Q2 (14-17 points), 23.16% (524/2263) in Q3 (18-22 points), and 22.36% (506/2263) in Q4 (23-55 points). The median CYPAT score in the total sample was 17 points (of a total possible of 55 points). There was a statistically significant (*P*<.001) difference between the median CYPAT score for those who attempted penetrative sex in the past 4 weeks (median 16) versus those who did not (median 18). Higher CYPAT scores were correlated with higher weekly exposure to pornography (*P*<.001; Figure 3).

**Figure 4:**
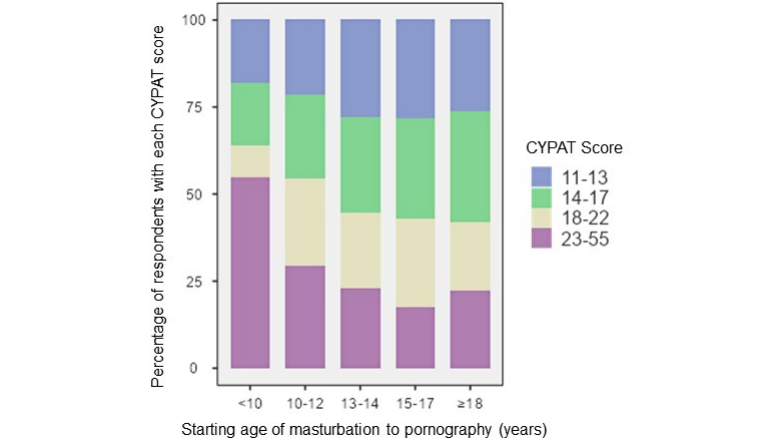
Correlation between Cyber Pornography Addiction Test (CYPAT) score and age at which masturbation to pornography started.

An earlier starting age was correlated with higher CYPAT scores (*P*<.001; Figure 4). In the group that started watching pornography below the age of 10 years old, >50% (12/22, 55%) had a CYPAT score in the 4th percentile of our population scoring range.

Of our participants, 21.61% (525/2429) indicated a need to watch an increasing amount of or increasingly extreme pornography to achieve the same level of arousal, and 10.39% (252/2425) needed to do this to get the same rigidity of their penis.

Of the sexually active participants, 72.74% (1238/1702) said they never had erectile, arousal, or climaxing difficulties in the previous 4 weeks when masturbating with porn, compared with 64.8% (456/704) of the sexually inactive men. Only 43.03% (756/1757) of the sexually active men had never had erectile, arousal, or climaxing difficulties when masturbating without porn, compared with 39.43% (289/733) of the sexually inactive men.

Of the sexually active men who were classified as having ED, 61.4% (213/347) admitted to never having erectile, arousal, or climaxing difficulties when masturbating with porn, versus 32.5% (115/354) when masturbating without porn.

#### Erectile Dysfunction

The following results are based on the participants who attempted sexual intercourse during the past 4 weeks (2067/3419).

According to their IIEF-5 scores, 21.48% (444/2067) of our sexually active participants (ie, those who attempted penetrative sex in the previous 4 weeks) had some degree of ED (mild: 77/444, 17.4%; mild-moderate: 15/444, 3.4%; moderate: 3/444, 0.6%; severe: 1/444, 0.2%). Most ED was mild (IIEF-5 score: 17-21). However, this mild ED bothered 61.2% (272/444) of affected individuals.

**Figure 5:**
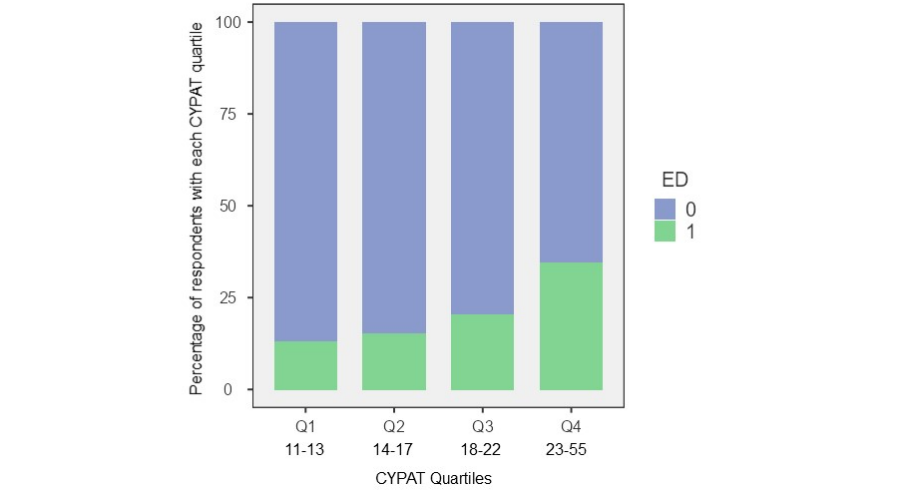
Correlation between Cyber Pornography Addiction Test (CYPAT) scores and erectile dysfunction (ED). 0=No, 1=Yes.

Regarding the correlation between PPC and ED, as shown in Figure 5, there was a statistically significant correlation between ED and CYPAT (*P*<.001). Higher CYPAT categories were associated with a higher prevalence of ED. Categorical analysis between the absence or presence of ED and CYPAT score showed a significantly higher median CYPAT score in the ED group (ED: median 19; no ED: median 16; *P*<.001). In men with the lowest CYPAT scores (Q1), only 12.9% (59/459) suffered from ED, increasing to 34.5% (127/368) in Q4. In the group who was sexually active and had a CYPAT score >28 (9th percentile), 49.6% (58/117) had some form of ED. When looking at the different ED categories based on the IIEF-5 scoring, there was a highly significant difference in CYPAT score (*P*<.001). Post hoc analysis showed a difference in median CYPAT scores between mild to moderate ED (median CYPAT: 24), mild ED (median CYPAT: 19), and no ED (median CYPAT: 16).

Of the participants classified as having ED, 27.7% (123/444) needed to watch more or more extreme pornography to achieve the same level of arousal, compared with 18.9% (84/444) in those participants who did not experience this need.

Of the participants who had started masturbating to porn at a very early age (<10 years), 58% (11/19) had some form of ED (*P*=.01), compared with 20.7% (61/295) in the group who started at 10-12 years old, 20.8% (173/831) in the group who started at 13-14 years old, 18.6% (97/521) in the group who started at 15-17 years old, and 24% (17/70) in the group who started at an age of 18 years or older.

**Figure 6:**
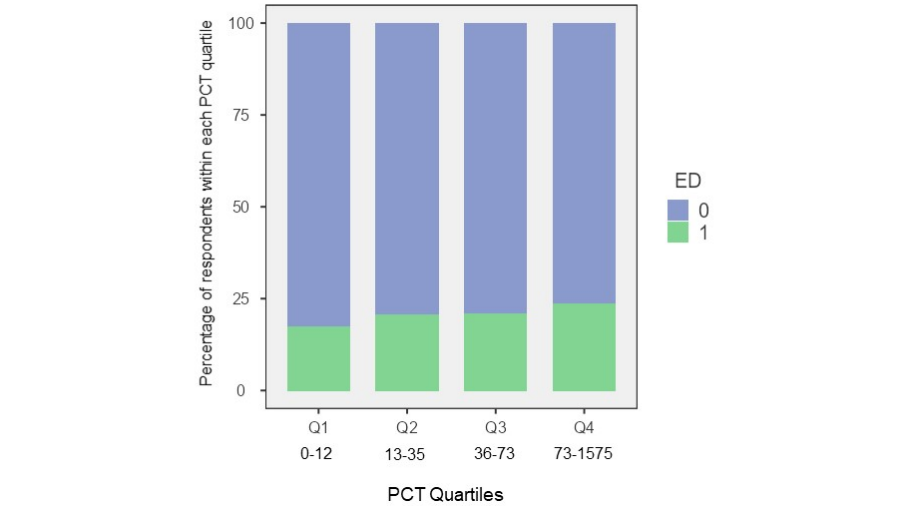
Correlation between pornography consumption time (PCT; minutes) and erectile dysfunction (ED). 0=No, 1=Yes.

Regarding the correlation between PCT and ED, we could not find a statistically significant correlation between ED and PCT when divided in quartiles (*P*=.17; Figure 6). However, in the group of participants with ED, the median time spent masturbating to porn was 39.81 minutes versus 31.50 minutes in the non-ED group, which was statistically significant (Kruskal Wallis 4.74; *P*=.029).

There was a statistically significant difference in the percentage of ED between people that frequently watched porn for more than 30 consecutive minutes (84/341, 24.6%) and those who did not (261/1330, 19.62%; *P*=.041). We did not find any correlation between ED and the number of opened videos per session

**Figure 7:**
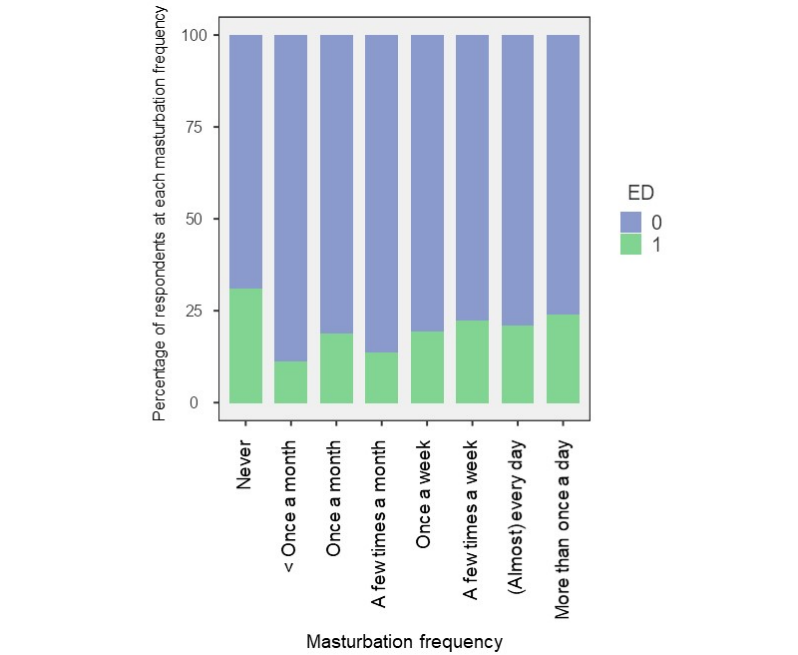
Correlations between masturbation frequency and erectile dysfunction (ED). 0=No, 1=Yes.

Regarding the correlation between masturbation frequency and ED, there was no statistically significant difference in masturbation frequency between the ED and no ED groups (*P*=.28; Figure 7), even when 3 categories of self-reported masturbation frequency were defined: low frequency: never to once a week, 388/1798, 21.58%; medium frequency: few times a week to every day, 1272/1798, 70.75%; and high frequency: regularly more than once a day, 138/1798, 7.68%. ED was found in 16.9% (65/384) of the low-frequency, 21.59% (266/1232) of the medium-frequency, and 23.9% (32/134) of the high-frequency groups. However, this difference was not statistically significant (*P*=.09). The median IIEF-5 scores in the low-frequency, medium-frequency, and high-frequency group were the same (ie, 24).

Regarding other possible confounders and ED, we observed a difference in self-reported presence of morning and spontaneous erections between those affected by ED (spontaneous: 344/404, 85.1%; morning: 375/404, 92.8%) and those not affected by ED (spontaneous: 1321/1474, 89.62%; morning: 1408/1474, 95.52%; *P*=.02).

There was a small but statistically significant (*P*<.001) difference in median self-reported libido score on a scale from 1 to 10 between those affected by ED and those who were not (7.4 vs 7.8).

In our study population, we could not find a correlation between AUDIT-C score and ED (median score was 5 in both groups). However, smoking was correlated with worse erectile function (active: 517/1966, 26.3%; occasionally: 497/1966, 25.3%; never: 411/1966, 20.9%; past: 332/1966, 16.9%; *P*=.020).

In the ED group, 17.4% (77/443) and 4.5% (20/443) said that most or a lot of their sexual contact happened under the influence of alcohol or drugs, respectively, versus 6.50% (99/1523) and 1.97% (30/1523) of those without ED (*P*<.001 [alcohol] and *P*=.003 [drugs]). For those who frequently had sex under the influence, >40% (77/176, 43.8%) were classified as having ED.

There was no significant correlation between the use of antidepressants and ED in our study population (*P*=.08).

Relationship satisfaction also seemed to be correlated with ED (*P*<.001): 10.8% (55/508) of people that were extremely satisfied with their relationship had ED versus 33% (10/30) of people that were extremely unsatisfied. However, this correlation did not seem to be linear.

### Problematic Pornography Consumption and ED in the Multivariate Analysis

A multivariable logistic regression model was built using the presence or absence of ED as a dichotomous outcome variable, taking exposure and other selected variables into account. CYPAT, performance pressure, and libido were considered as continuous variables (not normally distributed). Sexual identity, masturbation frequency, relationship status, real sex versus pornography preference, use of antidepressants, and partner satisfaction were considered as categorical variables. Table 3 contains an overview of the logistic regression model results.

There were 8 factors that were statistically significant (*P*<.05). The OR for CYPAT was 1.06 (95% CI 1.03-1.08; *P*<.001), which means that for each unit increase in CYPAT score, the odds of ED increase by 6%. Participants who answered having sexual fantasies about men only resulted in an OR of 2.66 (95% CI 1.52-4.66; *P*<.001) as compared with men having fantasies about women only. The OR for libido (“How would you rate your libido on a scale from 1 to 10?”) was 0.79 (95% CI 0.71-0.89; *P*<.001), which means that the odds of ED decrease by 21% for every unit increase in libido. Experiencing performance pressure (“Pressure to perform in bed or to maintain an erection while having sex on a scale from 1 to 10”) resulted in an OR of 1.30 (95% CI 1.24-1.38; *P*<.001). Being single or having a new relationship was found to raise the odds of ED (OR 2.12, 95% CI 1.34-3.36; *P*=.001 and OR 2.27, 95% CI 1.40-3.66, *P*<.001, respectively) as compared with men in a longstanding (>6 months) relationship. The OR for arousal (real sex vs pornography) was also found to be statistically significant, comparing men who reported that real sex gave them a lower level of arousal than pornography with men reporting the same level of arousal with real sex and pornography (OR 2.34, 95% CI 1.29-4.25; *P*=.005). There was also a significant difference between men who were extremely satisfied with their overall sexual relationship and men who were moderately satisfied (OR 0.58, 95% CI 0.39-0.86; *P*=.007). Masturbation frequency and the use of antidepressants were not statistically significant in this model.

**Table 3:** Logistic regression model coefficients and odds ratios for erectile dysfunction.

Predictor			Estimate^a^		SE		*Z*		*P*		Odds ratio		Lower 95% CI		Upper 95% CI
Intercept			–160.857		0.4993		–322.144		.001		0.200		0.0752		0.533
CYPAT^b^ score			0.05586		0.0122		456.757		<.001		1.057		1.0324		1.083
Performance pressure			0.26506		0.0271		977.692		<.001		1.304		1.2361		1.375
Libido (“How would you rate your libido?” [scale 1-10])			–0.23398		0.0579		–403.809		<.001		0.791		0.7064		0.887
**Masturbation frequency (Ref=a few times a week, not every day)**															
	Never	134.592		0.8864		151.842		.13		3.842		0.6761		21.829	
	Less than once a month	–0.88466		0.9204		–0.96113		0.34		0.413		0.0680		2.508	
	Once a month	–0.14011		0.6079		–0.23046		0.82		0.869		0.2640		2.862	
	A few times a month	–0.41440		0.3257		–127.235		0.20		0.661		0.3490		1.251	
	Once a week	0.13267		0.2592		0.51177		0.61		1.142		0.6870		1.898	
	(Almost) every day	–0.22186		0.1846		–120.164		0.23		0.801		0.5578		1.150	
	Regularly more than once a day	–0.06230		0.2866		–0.21741		0.83		0.940		0.5358		1.648	
**Use of antidepressants (Ref=No)**															
	Yes	0.71061		0.4453		159.573		0.11		2.035		0.8503		4.872	
**Partner satisfaction (“How satisfied are you with the overall sexual relationship you have with your main partner?”; Ref=moderately satisfied)**															
	Extremely satisfied	–0.55291		0.2038		–271.344		0.007		0.575		0.3859		0.858	
	Extremely unsatisfied	0.00388		0.5325		0.00729		0.99		1.004		0.3536		2.850	
	Moderately unsatisfied	–0.24571		0.2816		–0.87252		0.38		0.782		0.4504		1.358	
	Neither satisfied nor unsatisfied	–0.30223		0.2834		–106.627		0.29		0.739		0.4241		1.288	
**Sexual orientation (Ref=women only)**															
	Men and women equally	–0.85470		0.5628		–151.873		0.13		0.425		0.1412		1.282	
	Men mostly	0.58608		0.4662		125.715		0.21		1.797		0.7206		4.481	
	Men only	0.97824		0.2858		342.253		<.001		2.660		1.5190		4.657	
	Men somewhat more than women	0.29131		0.6834		0.42628		0.67		1.338		0.3506		5.107	
	Women mostly	–0.18640		0.2140		–0.87090		0.38		0.830		0.5456		1.262	
	Women somewhat more than men	0.57143		0.4021		142.123		0.16		1.771		0.8052		3.894	
**Relationship status (Ref=in a longstanding relationship [>6 months])**															
	Divorced/widowed	159.908		13.664		117.029		0.24		4.949		0.3399		72.036	
	Engaged/married	–0.13043		0.2211		–0.58989		0.56		0.878		0.5690		1.354	
	In a “new” relationship (<6 months)	0.81798		0.2448		334.148		<.001		2.266		1.4024		3.661	
	Single	0.75201		0.2346		320.566		.001		2.121*		1.3394		3.360	
**Arousal (Ref=real sex gives me the same level of arousal as pornography)**															
	Real sex gives me a higher level of arousal than pornography	–0.18049		0.1902		–0.94916		0.34		0.835		0.5751		1.212	
	Real sex gives me a lower level of arousal than pornography	0.84936		0.3043		279.130		0.005		2.338		1.2878		4.245	

^a^Estimates represent the log odds of “ED” vs “No ED.”

^b^CYPAT: Cyber Pornography Addiction Test.

## Discussion

### Principal Findings

Since 2006, with the rise of so-called “porn tube sites,” pornography has become widely available and easily accessed on the internet. With just a click, the consumer can indulge his fantasies in ways that would never be possible in real life. Although our study was not intended to examine pornography consumption habits in the general population, the high pornography consumption rates in our study were similar to those in several population studies [47,48] and in line with Pornhubs’ 2019 statistics [49]. While pornography-assisted masturbation is more frequent nowadays, this is not necessarily a sign of pathology [50]. Masturbation with pornography is even a source of sexual health for many young men.

However, since many men start using pornography at a very early age and masturbate more with the help of pornography than without, it is important to study its possible consequences on erectile function.

Frequency of pornography use did not seem to have an important impact on the occurrence of ED. Only when consuming pornography for more than 30 minutes in a row was the frequency of ED slightly higher, but most participants (89%) do not consume pornography for more than 30 minutes.

For the multivariate analysis, a DAG was used to guide the multivariable data analysis to avoid inappropriate adjustment for variables on a causal path between exposure and outcome. Our DAG might be biased since the associations between the covariates are not well known. We hypothesize that the DAG is in proximity of the truth since it was based on the best available evidence and multidisciplinary subject matter expertise when evidence was not available. Age, a well-known covariate for ED, was not included in the DAG because the effect of age was not considered important in our target population (≤35 years of age). Alternative logistic regression models including other variables such as “duration of one pornography session,” “masturbation ratio with and without pornography,” and “whether pornography is needed to climax” were examined but had lower performance and resulted in a poor model fit compared with the model based on the DAG. Among the model’s covariates, there was little multicollinearity, and no extreme influential observations were detected, thus meeting the assumptions for logistic regression.

More PPC, as measured by CYPAT in our study, resulted in a higher probability of ED, while controlling for covariates. While an OR of 1.06 seems low, it is important to remember that for each increase in CYPAT score, the chance of ED increased by 6%. As the CYPAT consists of 11 questions and for each question, a score of 1-5 is given, it means that when 3 questions are scored 1 point higher, the odds for ED increase by 18%, which is high. While a longitudinal study will be necessary to draw conclusions, it is striking how many young men who are not having sexual intercourse (yet) have high CYPAT scores.

There was a wide variety of PCT for different CYPAT categories, meaning that the time of pornography consumption is not necessarily predicting CYPAT scores and vice versa.

Two other instruments are recommended to assess PPC [36]: the Problematic Pornography Use Scale and Problematic Pornography Consumption Scale (PPCS). However, they were published after we developed our survey. The PPCS should be considered in future studies as it has a clear cut-off for problematic versus nonproblematic use and furthermore assesses tolerance. However, CYPAT includes a use despite harm component, which is a relevant CBSD criterium. A correlation study between both scores would be relevant.

Masturbation frequency is often seen as a confounding factor when examining pornography consumption and relational happiness [3], as pornography consumption is mostly accompanied by masturbation. However, in our study, we found no evidence to support that masturbation frequency has an effect on ED. Masturbation frequency was not statistically significantly different between the ED versus no ED groups. Although men with ED watch more pornography per week, when examining the effect of CYPAT on ED, there was no significant effect of masturbation frequency.

ED was reported more frequently by men identifying themselves as homosexual and by men who had sexual fantasies about other men, but not necessarily identifying themselves as homo- or bisexual. Janssen and Bancroft [10] also documented a higher prevalence of ED among homosexual men in 2007. Sexual orientation, identity, and sexual fantasies towards other men seem to be important covariates when assessing the effect of PPC on ED. We also noticed that there are discrepancies between how men identify themselves and who their fantasies are about. Men who are sexually oriented to other men watch more pornography, have higher CYPAT scores, and report more ED. A follow-up study is being planned to understand the role of sexual identity, sexual orientation, and sexual fantasies on PPC and ED and how these covariates relate with each other.

Single men and men in a new relationship reported more ED than men in a longstanding relationship. Performance pressure, anxiety, and insecurity are important factors to assess when a young man consults for ED. Finding pornography more arousing than real sex also contributes to this situational ED (56% ED in our study sample versus 17% for those who found real sex more arousing). This was also seen in a study by Berger et al [51]. In his study, 79% of participants who preferred pornography over partnered sex were classified as having ED. Whether this is due to the incongruence between the participants who preferred pornography category or sexual preference and the partnered performance or if certain pornography categories are more prone to be more arousing needs to be studied further. However, it seems interesting to question pornography watching habits, including which pornography categories men find more arousing and how these relate to their own sexual practices.

One of the strengths of our study is that we assessed ED with a validated and broadly used scale with well-defined cut-off values to evaluate ED in a clinical urological context. However, many participants who did not have intercourse in the preceding 4 weeks were excluded from the analysis, as the IIEF questions were related to sexual intercourse during this period. In that sense, a questionnaire evaluating (situational) ED in young men based on questions not relating to sexual intercourse would be of great value. The ED part of the Male Sexual Health Questionnaire seems promising; however, no cut-off values are available as of yet. Also, the newly developed Masturbation Erection Index could be of great value [52].

On the other hand, it is clear that the ED seen in our study is situational, as many participants experiencing some ED during partnered sex did not experience ED nor climaxing difficulties while masturbating with pornography. In a clinical setting, while questioning a young patient presenting with ED, it can be interesting to question erectile function while masturbating with and without pornography consumption separately.

It should be said that our results are based on a survey sample. As is seen with the association between frequency of pornography use and problematic pornography use, it is possible that the association between PPC and ED could even be stronger in a clinical sample of treatment-seeking individuals [4].

Therefore, we should not wait to assess PPC in young males consulting for ED. Earlier studies showed only 3%-4.4% of men will consider themselves addicted to pornography [48].

However, most men experiencing ED possibly due to PPC cannot be considered as “addicted.” The prevalence of ED already significantly increases with moderate CYPAT scores. Is it possible this situational ED is an early warning signal for the impact pornography has on their sexual functioning?

As pornography consumption is common nowadays (and is even growing, with 11% increased global traffic during the COVID-19 pandemic [53]), most young men will not bring up the topic themselves, and if health care professionals treating ED do not assess pornography consumption in a structured way, the impact in the clinical setting will not be known. As long as this impact is not known, even if it is only self-perceived, it will be impossible to agree on diagnostic criteria and possible treatment algorithms, keeping many individuals in a vicious circle affecting quality of life, possibly having an adverse effect on a man’s psychosocial well-being, and placing a burden on relationships [15].

While the existence of “porn addiction” is disputed, there seems a strong correlation between CYPAT and ED. We found an OR of 1.06 for every point increase in CYPAT scores and ED. Indeed, to this day, “PIED,” “porn addiction,” and “sex addiction” do not exist as diagnosable entities in the DSM-5. On the other hand, very recently, the World Health Organization included a new related diagnosis to their ICD-11, CSBD, under which compulsive use of pornography could be classified. It was categorized as an impulse control disorder and not as a behavioral addiction because there is insufficient evidence to do so [36].

Of course, further neuroscientific and psychophysiological studies will be necessary to explain why PPC can have an effect on ED. To achieve tumescence, a man needs sexual arousal, and this may come from visual stimulation. With sufficient arousal, nitric oxide is released in the penile cavernous tissue and the GTP-cGMP-5’GMP cascade is started. This is the physiological target for PDE-5 inhibitors that are commonly used to treat ED. In the absence of arousal, there will be no erection. A hypothesis is that pornography may give such an extreme visual stimulus that it overactivates the reward system in our brains [21,22]. As with other addictions, the brain rewires itself and accommodates this overstimulation; thus, more and more extreme porn is required to achieve the same level of arousal (tolerance), to the point where normal sex with a partner is no longer sufficient for arousal. Although not validated, this hypothesis will implicate that PDE-5 inhibitors commonly used to treat ED will be less effective in patients suffering from PIED.

As our study shows a higher ED rate in those who started consuming pornography at an earlier age, we need to learn more about adolescents’ pornography use in a larger social and cultural development context [54] and consider media effects [55]. Also, we need to focus on longitudinal studies to examine the effects of early exposure, as a Croatian study already found that higher baseline levels of pornography use as well as higher levels of negative emotions and impulsivity predicted higher levels of PPC 3 years later [56]. This should also trigger more interdisciplinary work on this topic with medical specialists, sexologists, (developmental) psychologists, sociologists, and specialists in media literacy. Next, we should focus on porn literacy programs. A recently developed digital prototype seems promising to address the pornography literacy needs of young people [57].

### Strengths and Limitations

We consider the large number of observations, the use of validated scales, the multidisciplinary approach, and the multivariable analyses based on a DAG as the major strengths of this study. Although the study was conducted with great care, possible biases might have been introduced. The study sample might not be fully representative of the population intended to be analyzed. Men with sexual health problems might have been more prone to participate in the study, resulting in a higher prevalence of ED in the study sample. Measurement bias due to using CYPAT as a (initially unintended) measure for PPC may also be present. It is also possible that recall bias was present caused by differences in the accuracy of the recollections retrieved by study participants regarding past behavior, especially when estimating the PCT per week. Also, one of the big problems in this field of research is that there is basically no control group, since nearly all young men seem to watch pornography during masturbation.

The association we found between PPC and ED does not necessarily mean that PPC causes ED. It is perfectly possible that ED leads to higher levels of pornography consumption. However, our multivariable analysis was based on a DAG model that included ED as outcome parameter, not the other way around. This suggests a possible causal association, although more research is needed to investigate causality in depth. Also, DAGs are usually used to encode a priori assumptions about relationships between variables to express causal assumptions and to guide the data collection and analysis [58]. Here, a DAG was used a posteriori.

### Conclusions

The prevalence of ED in young men is alarmingly high, and the results of this study suggest a significant association with PPC. Higher CYPAT scores result in a higher probability of ED, when controlling for covariates. Masturbation frequency is not a significant factor when assessing ED. Multivariable analysis identified sexual orientation, experiencing performance pressure, and relationship status as important factors when evaluating the effect of PPC on ED in young men.

## Abbreviations

AUDIT-C: Alcohol Use Disorders Identification Test-Concise
CSBD: compulsive sexual behavior disorder
CYPAT: Cyber Pornography Addiction Test
DAG: directed acyclic graph
DSM-5: Diagnostic and Statistical Manual of Mental Disorders, Fifth Edition
ED: erectile dysfunction
ICD-11: International Classification of Diseases, 11th Revision
IIEF-5: International Index of Erectile Function, Short version
OR: odds ratio
PCT: pornography consumption time
PIED: porn-induced erectile dysfunction
PPC: problematic pornography consumption
PPCS: Problematic Pornography Consumption Scale
